# The Crisis in the British Medical Association

**Published:** 1907-08-31

**Authors:** 


### The Hospital

A Journal of

The Medical Sciences and Hospital Administration.

New Series. No. 23, Vol. I. [No. 1095, Vol. XLII.] SATURDAY, AUGUST 31, 1907.

### THE CRISIS IN THE BRITISH MEDICAL ASSOCIATION.

Events for some time past have been suggesting that a crisis in the position and development of the British Medical Association was imminent, and now, it would appear, the issue is fairly joined. Two of the controlling organisations within the scheme of the Association have directly challenged one another, and it rests with the individual members in their several constituencies to say which of the two shall be supreme. It is inevitable that the controversy must be an acute one, and a good deal of strong feeling and strong speaking may be expected. But it will, we hope, be recollected by the disputants that the one question of importance is the welfare of the Association with which they are connected, and that this welfare is impossible unless the Association commands the support and sympathy of the vast majority of the profession. An active and energetic minority might, it is conceivable, score an apparent victory, but such victory would certainly be short-lived. The majority of medical practitioners hardly concern themselves in any active sense with professional politics, but they will not be slow to express their resentment should any Association, professing to speak on their behalf, assume an attitude which they regard as unworthy of the highest traditions of the profession. This aspect of the coming struggle cannot be too strongly emphasised. In the last resort the general opinion of the whole profession will prevail, and unless the Association's policy is framed in accordance with this, very serious consequences will ensue. At present, the Association is the one organisation through which unity and common action in the profession are possible. It would, in our judgment, be a profound misfortune were anything to limit its capacity for usefulness. We therefore venture to urge on all its members the need for careful consideration of the issues which are now to be presented for their consideration, and the vital importance of the decision which rests in their hands.

The crucial question in the impending struggle is briefly this—whether the Council of the Association or the Representative meeting shall be the supreme and final authority. At the recent meeting at Exeter, the last-mentioned body came to certain decisions in reference to the clauses of the proposed Royal Charter, and the resolutions embodying these decisions were submitted in due course to the Council. From certain of these resolutions the Council has now formally indicated its dissent, and has appealed to the Divisions to disapprove of the proposals of the Representative meeting. The Council has, in short, subjected these proposals to the Referendum, so that the issues involved must be fought out by the members of the Association as arranged in Divisions, and the ultimate decision must rest with the majority. The question, however, is whether the opinion of an absolute majority of the members will be obtained. Everyone must admit that this is desirable, or even essential, but will the existing machinery secure it? By that machinery the resolutions of the Representative meeting challenged by the Council will be submitted to the Divisions, and a special meeting of each of these will be summoned to consider them. But it is notorious that, at least in many of the Divisions, any such meeting secures but a scanty attendance, and a vote there taken may readily fail to represent the real opinion of the constituency. Of course, it may be said that members ought to attend, but the fact remains that they do not. Therefore, if the votes which are to determine the issue are to be solely those recorded in the Divisional meetings, there is a real risk that the true feeling of the profession will not be obtained. And this, as we have already pointed out, implies a position which is not free from danger to the Association. The desirable thing would be the substitution of voting papers issued to all members of the Association, instead of a restriction of the votes to those recorded in the Divisional meetings. In this way the result would be not only conclusive, but it would be relatively free from danger. No one could challenge the extent of the opportunity, and there would not be the risk of the Association formally adopting a position for which the moral support of the majority of its members did not exist. Whether the substitution of the voting paper for the Divisional meeting is within the bounds of the Constitution or not we cannot pretend to say, but that such substitution is desirable if the Association is to occupy a safe and confident position there can be little doubt. Certainly, in the last resort, the voice of the majority must prevail. But let care be taken to see that the majority is a real and not an accidental one.

Concerning the merits of the issue to be submitted we have at present nothing to say. But the comments already applied to the mode of voting on the Referendum apply almost equally to the Representative meeting. The decisions of that meeting may be wise or unwise, but they are wanting in moral strength, owing to the fact that so few of the members of the Association take part in the election of the representatives. Such elections take place in the Divisional meetings, where so often, as already stated, the attendance of members is of the scantiest. How, for example, can a representative claim to speak for several hundred members when the actual votes recorded in his favour scarcely, perhaps, reach the dignity of double figures? We are not in the least criticising the decisions of the Representative meeting; but until the state of matters just mentioned is altered, these decisions have little claim to be the accredited voice of the Association.

